# Immobilization and enhancement of a heterodimeric fluorescence biosensor in fibrous protein biomaterials

**DOI:** 10.1002/pro.70119

**Published:** 2025-04-22

**Authors:** Rebecca M. Booth, Amanda Jons, Xue Gong, Shounak Banerjee, Britt Faulk, Hays Rye, Christopher Bystroff, Sarah E. Bondos

**Affiliations:** ^1^ Department of Molecular and Cellular Medicine Texas A&M Health College of Medicine Bryan Texas USA; ^2^ Interdisciplinary Graduate Program in Genetics Texas A&M University Texas USA; ^3^ Department of Biochemistry and Biophysics Texas A&M University Texas USA; ^4^ Department of Biological Sciences Rensselaer Polytechnic Institute Troy New York USA; ^5^ Department of Medical Physiology, School of Medicine Texas A&M University, College of Medicine Bryan Texas USA

**Keywords:** Hox transcription factors, protein fusion, protein‐based materials, split complementation assay

## Abstract

Leave‐one‐out green fluorescent proteins (LOO_GFPs) have a reduced quantum yield relative to the parent protein and form fluorescent oligomers in the unbound state. Immobilizing LOO_GFPs in materials composed of the *Drosophila* protein Ultrabithorax (Ubx) via gene fusion increased the fluorescent signal, significantly stabilized the biosensor, and prevented oligomerization into fluorescent aggregates, which has the potential to elevate the sensor's noise well above the signal. Interactions between LOO_GFP and Ubx hampered analyte rebinding. By optimizing the concentrations of LOO_GFP, salt, and detergent in the assay, the signal to noise ratio for the biosensor increased fourfold. These modified fibers represent the first incorporation of a protein complementation assay into protein‐based materials, as well as the first incorporation, via gene fusion, of a heterodimeric functional protein into materials composed of a different self‐assembling protein. This study highlights the advantages and identifies potential pitfalls associated with protein immobilization in materials.

## 1 INTRODUCTION

Biosensors are devices that rely on living organisms and/or biological molecules to detect specific substances or conditions, often disease agents or environmental contaminants (Jarczewska & Malinowska, 2020). Biosensors must specifically recognize an analyte and transduce a signal upon analyte binding that ideally can be detected using simple, portable equipment. Furthermore, biosensors must be biocompatible if used in vivo. Using proteins as biosensors offers a unique opportunity to achieve these goals: proteins can bind other molecules with unparalleled specificity, they can be engineered to recognize different analytes, they can create visible, electronic, or chemical signals, and they are a natural component of all life (Calabrese et al., 2023; Dong et al., 2023; Liang et al., 2019; Ménard‐Moyon et al., 2020; Quijano‐Rubio et al., 2021; Sadeghzadeh et al., 2023).

Protein‐fragment complementation assays (PCAs) provide a mechanism to link analyte binding with signal generation within a single protein. In this approach, a protein is divided into two chains which individually lack function but, when recombined, reconstitute the protein structure and generate a signal. Many PCAs have been designed to investigate protein–protein interactions in a wide variety of fields (Romei & Boxer, 2019, Nguyen & Silberg, 2010). These assays use a variety of readouts, including fluorescence, bioluminescence, cell survival, and gene transcription (Romei & Boxer, 2019). In particular, split green fluorescent protein (GFP) systems offer the advantage of a self‐reporting fluorescent readout that requires no reagents. Thus, on‐site detection and analysis is both possible and facile, requiring only a blue light source and an orange high‐pass filter, which can be adapted for use with a standard smart phone.

**FIGURE 1:**
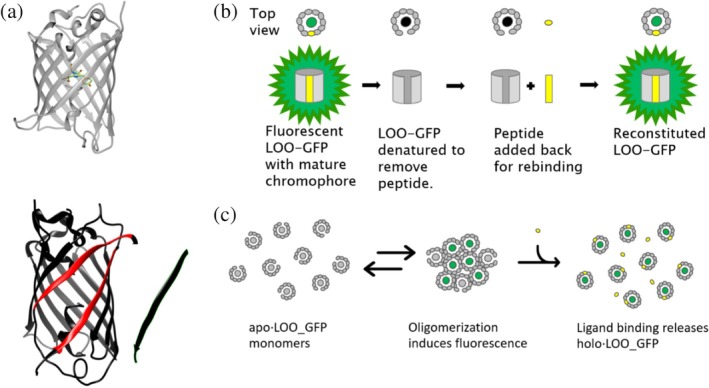
LOO_GFP design and oligomerization. (a) Ribbon diagram of OPT_GFP rotated to visualize the chromophore (PDBID: 2b3p) shown above an illustration of LOO8 without the chromophore. The cavity in LOO8 created by removal of the eighth strand is highlighted in red. The left‐out peptide (s8) is shown to the right. (b) Overview of LOO8 processing. LOO8 is co‐expressed with its left‐out peptide (shown in yellow), termed the “priming” peptide, to ensure chromophore maturation, proper folding, and fluorescence (green starburst). Denaturing conditions remove the priming peptide to stop fluorescence. Addition of synthetic “target” peptide allows rebinding and reconstitutes fluorescence. Throughout this paper, the priming and target peptides have the same amino acid sequence (Table S1). (c) Monomers of LOO7 and LOO8 (discussed herein) oligomerize, causing high background fluorescence and reducing the signal‐to‐noise ratio (Y.‐M. Huang & Bystroff, 2009). Ligand binding disrupts the auto‐fluorescing oligomers. GFP, green fluorescent protein; LOO, leave‐one‐out.

GFP fluoresces via a chromophore, generated by post‐translational modification of three residues which lie at the center of an 11 β‐strand barrel (Figure 1a, top) (Crone et al., 2013). The leave‐one‐out split GFP (LOO_GFP) system was engineered from OPT, a variant of superfolder GFP that has been optimized for higher solubility (Cabantous et al., 2005). In LOO_GFPs, the protein sequence is circularly permuted and truncated to omit one or more of the 11 β‐strand segments (Figure 1a, bottom) (Y.‐M. Huang, Nayak, et al., 2011). In principle, when a strand is removed, the chromophore is free to internally rotate and does not emit light (Megley et al., 2008). Binding by the excluded GFP peptide strand completes the protein structure, immobilizes the chromophore, and restores fluorescence (Y.‐M. Huang & Bystroff, 2009). The excluded portion thus becomes the peptide analyte, which restores fluorescence when this peptide binds the LOO_GFP, thus activating the sensor. This approach unites sensing and reporting in a single molecule. Ultimately, the residues surrounding the gap in LOO_GFPs can be re‐designed to bind and detect peptides of a desired sequence in lieu of the original left‐out peptide sequence (Figure 1a). Indeed, LOO7 has been redesigned to detect the HA protein from influenza (Y.‐M. Huang et al., 2015) and the Aβ motifs (Yu et al., 2023). Because using LOO_GFP sensors requires little equipment, in the long term these redesigned sensors could be deployed in remote locations or in field studies, such as searching for viruses in mosquito vectors.

Because only a small segment is left out of LOO_GFPs, most of the folding pathway is retained and the unbound LOO_GFP folds to a near‐native structure, ready to bind the peptide and complete the formation of the β‐barrel. Even so, chromophore maturation can require several hours (Cabantous et al., 2005; Romei & Boxer, 2019). Consequently, the LOO_GFP is typically co‐expressed with a “priming” peptide, typically the same amino acid sequence as the left‐out peptide. This priming peptide must be removed to empty the binding site and allow the analyte to bind (Figure 1b). LOO_GFP variants are named after the removed strand. For instance, in LOO7, the seventh strand is removed and the corresponding peptide, s7, functions as the analyte.

In previous work, LOO7 and a designed influenza biosensor, LOO7‐HA4, were found to form oligomers of variable size in the unbound state (Figure 1c). These oligomers fluoresce in the absence of their respective target peptides, substantially elevating the background signal (Huang & Bystroff, 2009, Kodama & Hu, 2012). Oligomerization also acts as a nonspecific competitor for LOO‐peptide binding (Hassibi et al., 2007). This problem has been reported for other GFP‐based biosensors as well (Romei & Boxer, 2019). The effects of nonspecific binding can generally be reduced by raising the concentration of the biosensor and target analyte relative to the nonspecific binder (Hassibi et al., 2007). In the case of oligomerization, the nonspecific binder is the biosensor, and increasing biosensor concentration would increase oligomerization. Conversely, lowering the biosensor concentration to reduce oligomerization would also reduce the maximum signal that can be detected. Thus, the problem cannot be easily solved by adjusting the concentrations of the components. Moreover, split fluorescent proteins typically have a lower quantum yield than the original protein due to a less stable β‐barrel structure, further reducing the maximum obtainable signal (Köker et al., 2018). In the case of LOO7, the quantum yield for LOO7 bound to its peptide s7 (s7:LOO7) was only 40% of that of the parent protein OPT_GFP (Huang & Bystroff, 2009).

**FIGURE 2:**
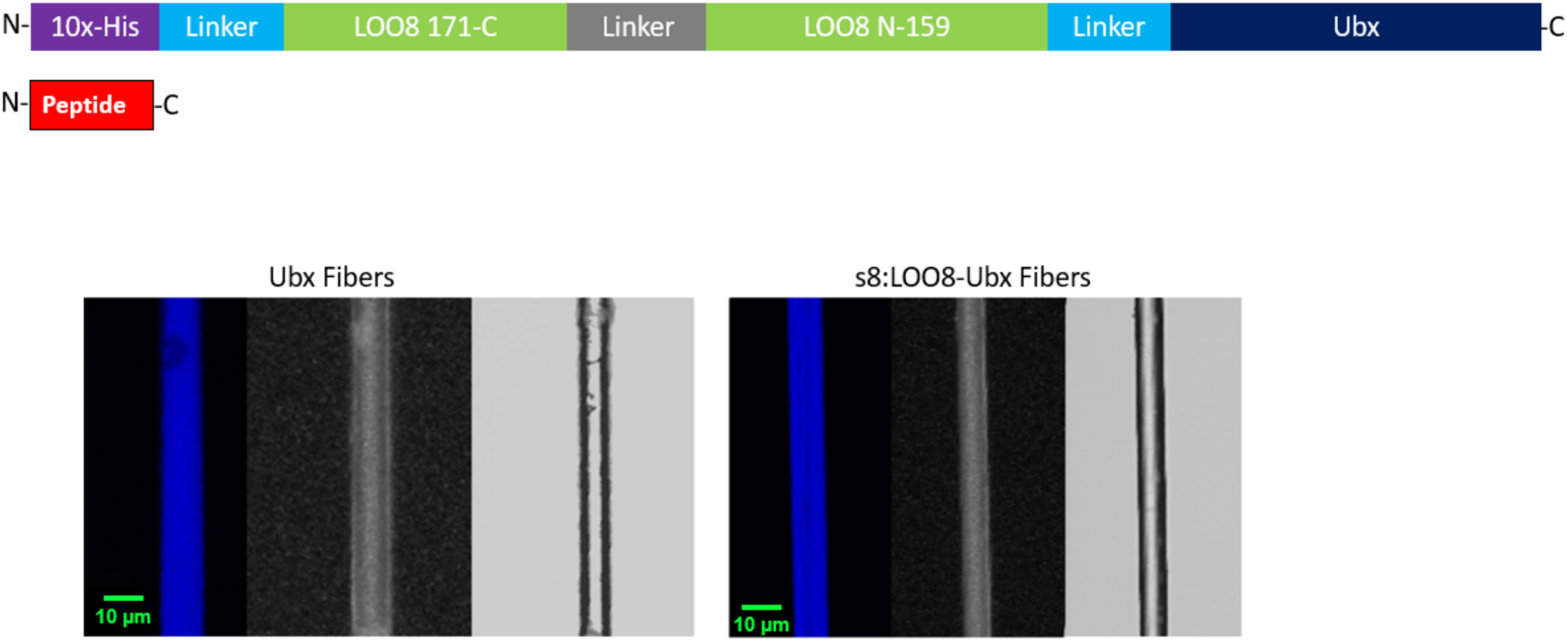
Inclusion of LOO8 does not harm Ubx fibers. (Top) A schematic of the LOO8‐Ubx fusion protein including the N‐terminal His‐tag (purple), the circularly permuted and truncated LOO8 protein (green), whose two halves are connected by a linker amino acid sequence (gray), Ubx (dark blue), and glycine/serine‐rich linkers (light blue). The untagged s8 peptide NGIKANFTVRHNV corresponding to OPT_GFP residues 159–171 is shown separately (red). The LOO8 sequence includes amino acids 172–238, an artificial linker (GGTGGS), followed by amino acids 1–158 of OPT_GFP. All protein sequences are listed in Table S1. (Bottom right) Comparison of plain Ubx fibers (left) and s8:LOO8‐Ubx fibers (right) acquired using confocal microscopy to track blue fluorescence emanating from dityrosine bonds (left of each group), differential contrast microscopy (middle of each group), and light microscopy (right of each group). Like plain Ubx fibers, the s8:LOO8‐Ubx fibers lacked pits, thin segments, or fraying, features which would have indicated poor assembly (Z. Huang et al., 2010; Majithia et al., 2011). Scale bar is 10 μm for all images. Brightness and contrast of images were adjusted equally to enhance image quality. GFP, green fluorescent protein; LOO, leave‐one‐out; Ubx, Ultrabithorax.

Our proof‐of‐concept study addresses these problems by immobilizing a LOO_GFP biosensor within protein materials. When eventually deployed, immobilizing the biosensor can facilitate separating the sensor from contaminants in a sample that may obscure the results during the loading and washing steps, and thus simplify detection. LOO8 was chosen as our model due to its high solubility and its ability to express well when genetically fused to the materials‐forming protein, *Drosophila melanogaster* Ultrabithorax (Ubx). In mild buffers, Ubx self‐assembles into robust, biocompatible, non‐immunogenic materials at the air‐water interface (Greer et al., 2009; Patterson et al., 2013, 2014). The resulting materials are stabilized by spontaneously forming dityrosine bonds (Howell et al., 2015). Ubx can uniquely incorporate a wide range of active functional proteins into materials through gene fusion. In this approach, adding the gene encoding the functional protein adjacent to the gene encoding Ubx will result in a fusion gene. This fusion gene produces a single polypeptide, termed the fusion protein, which is composed of both the functional protein and Ubx (Figure 2a) (Howell et al., 2015; Z. Huang, Salim, et al., 2011; Tsai et al., 2015). A short, flexible amino acid linker is also inserted between the two proteins to allow independent folding and function. Subsequent self‐assembly of this fusion protein incorporates the fused functional protein into the materials. Thus far, all Ubx fusion proteins tested are able to both self‐assemble into materials and retain the function of the appended protein (Howell et al., 2015; Mendes et al., 2024; Tsai et al., 2015). This approach provides a very high concentration of functional protein in the materials, resulting in a very strong fluorescent signal.

This study highlights how interactions between a genetically fused functional protein and its self‐assembling host protein can have both positive and negative consequences. Surrounding the LOO_GFP with protein materials provides an opportunity to sterically block nonspecific LOO_GFP oligomerization, reducing the background noise of the sensor. Ubx materials also substantially stabilize the LOO_GFPs, allowing room temperature storage. Additionally, unassembled, soluble LOO8‐Ubx monomers exhibit significantly increased fluorescent signal compared to LOO8 without Ubx. By increasing the signal and decreasing the noise, immobilizing LOO8 significantly improves the signal‐to‐noise ratio relative to monomeric LOO8. Finally, for effective use as a biosensor, analyte peptides must access the target peptide binding site. However, we discovered that the unbound state of LOO8 interacts with other fiber components to block analyte binding. To solve this problem, we identified buffer conditions that mitigate this interaction, allowing LOO8 to bind its target peptide. In this first‐generation LOO‐Ubx biosensor, the fiber diameter, percent of fused monomers, and buffer conditions all influence both the signal and background levels. These studies identify both critical benefits and potential pitfalls of immobilizing biosensors. Furthermore, incorporating LOO8, noncovalently bound to its peptide and analyte (s8) in Ubx materials, is the first time a functional, heterodimeric protein has been incorporated into protein biomaterials via gene fusion.

## 2 RESULTS AND DISCUSSION

### 2.1 Verifying the structure and function of LOO8‐Ubx materials

Protein complementation assays based on GFP provide a convenient mechanism to unite analyte detection and signal generation in a single molecule. However, the resulting LOO_GFP biosensor proteins can oligomerize and fluoresce in the absence of the target peptide, increasing the background signal (Figure 1c) (Huang et al., 2015). Physically holding the LOO_GFP domains apart by covalently tethering them to a solid support immobilizes LOO_GFP and is expected to increase the average separation during analyte binding and removal. Thus, we expect immobilization should reduce unwanted LOO8 oligomerization.

We chose to immobilize LOO8 in materials composed of Ubx protein, because these materials substantially stabilize proteins appended via gene fusion (Tsai et al., 2015). The *loo8* gene was fused to the *ubx* gene via a sequence encoding a flexible linker (Figure 2a and Table S1). This protein fusion was co‐expressed with the left‐out strand 8 priming peptide (s8) to complete the GFP structure and enable chromophore maturation (Barondeau et al., 2003). To examine whether fusion to Ubx alters the activity of LOO8, we compared the quantum yield, relative to the GFP OPT standard, for LOO8 and LOO8‐Ubx. Quantum yield was calculated as counts‐per‐second per concentration of the chromophore. The differences were small but significant. The quantum yield of LOO8 was 90% ± 2% relative to its parent protein GFP OPT, whereas the quantum yield of LOO8‐Ubx was 106% ± 4% relative to GFP OPT. Identical results were obtained for two different samples. While creating LOO8 does not result in as much loss of fluorescence as LOO7, the quantum yield is still lower than the parent protein. This deficit appears to be rectified by fusion to Ubx. This may be due to the rigidification of LOO8 by Ubx binding, or caused by the new electrostatic environment presented by Ubx (Diaspro et al., 2006). Indeed, the quantum yield is sensitive to electrostatics due to charge separation in the excited state, but not the ground state (Bravaya et al., 2011).

The heterodimeric fusion protein complex (s8:LOO8‐Ubx) was purified and assembled into fibers (Figure 2b). Because attaching LOO8 to a self‐assembling protein has the potential to alter the properties of one or both proteins, it is necessary to assess whether LOO8 function alters the assembly or structure of the materials, as well as whether embedding LOO8 in materials alters biosensor function.

To test whether fusion to LOO8 hampered fiber assembly, we compared the assembly of LOO8‐Ubx to that of plain Ubx and enhanced green fluorescent protein (EGFP)‐Ubx. The LOO8‐Ubx fusion protein, bound to the s8 peptide, successfully self‐assembled into films that were then drawn into green fluorescent fibers. The timescale for materials assembly (10 min) matched that for both plain Ubx materials and EGFP‐Ubx materials. For the same protein concentrations, the size of films and thus the length and diameter of fibers composed of LOO8‐Ubx are similar to that of Ubx and EGFP‐Ubx, suggesting that the protein's assembly rate is unchanged, as previously demonstrated for Ubx and EGFP‐Ubx (Tsai et al., 2015). We next examined the morphology of the fibers, since conditions that restrict Ubx self‐assembly may also damage fiber structure. The morphology of the s8:LOO8‐Ubx fibers was typical for Ubx materials as indicated by microscopy (Figure 2b). s8:LOO8‐Ubx fibers have a uniform diameter, no pits or defects, and fluoresce blue due to dityrosine bond formation, similar to Ubx fibers (Greer et al., 2009) and materials composed of other Ubx fusion proteins (Tsai et al., 2015). The normal appearance of these fibers and their ability to self‐assemble on a typical timescale further suggests that fusion to LOO8 did not impact Ubx assembly.

**FIGURE 3:**
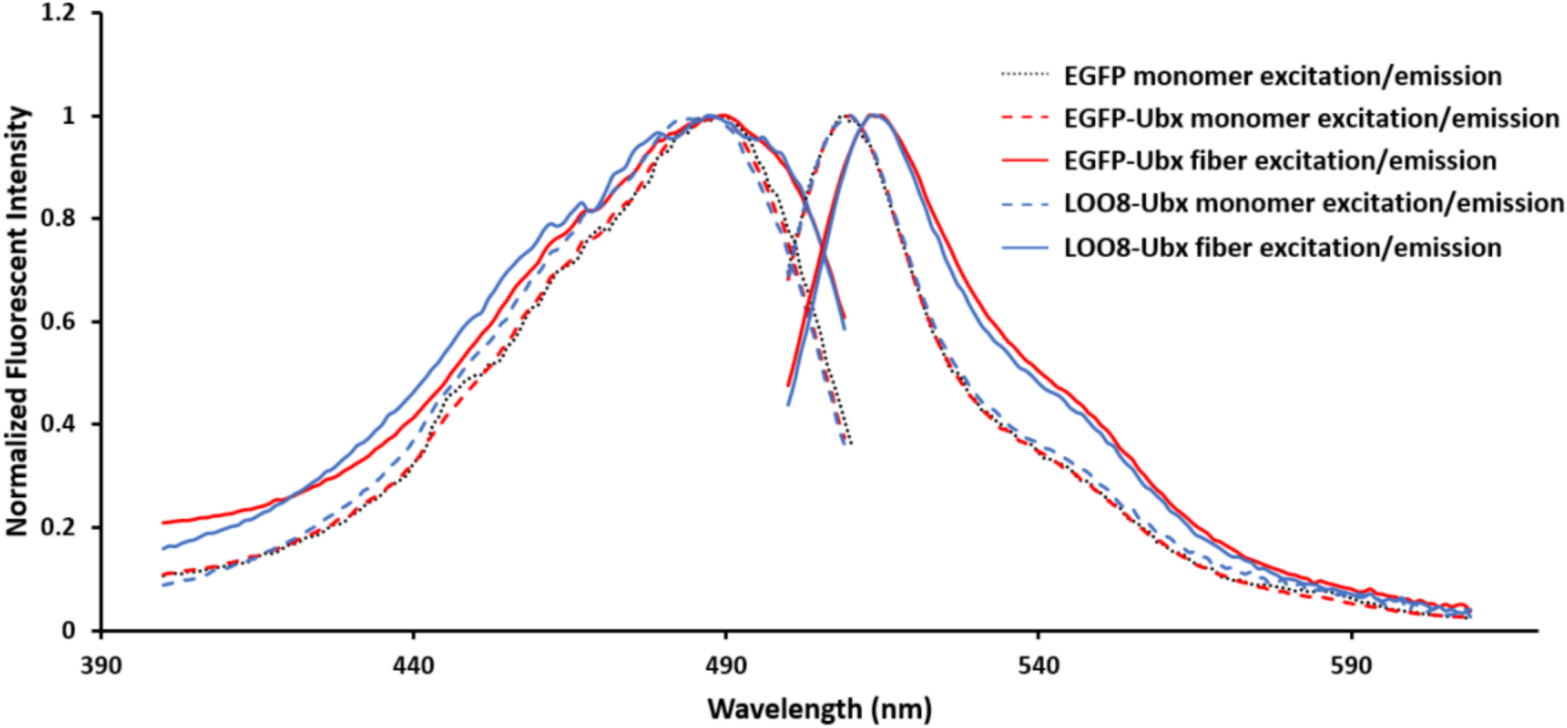
Spectra of LOO8‐Ubx are similar to EGFP‐Ubx. All samples had a maximum excitation peak at 488 nm, whereas the emission peak for all monomer samples (monomers of EGFP, EGFP‐Ubx, and LOO8‐Ubx as well as EGFP‐Ubx and LOO8‐Ubx fibers) had a maximum of 510 nm, and the emission peak for all fiber samples had a maximum of 515 nm. Spectra of monomers are reported using dotted or dashed lines, whereas fiber spectra are represented by solid lines. Peak maxima were normalized to 1 to compare the shape and position of spectra. EGFP, enhanced green fluorescent protein; LOO, leave‐one‐out; Ubx, Ultrabithorax.

Conversely, fusion to Ubx and assembly into materials could impact LOO8 function. Changes in LOO_GFP protein structure can alter the chemical environment and/or dynamics of the fluorophore, changing the shape or position of its emission spectrum (Huang et al., 2015). To determine whether Ubx fusion alters LOO8 function, we compared the fluorescence spectra of s8:LOO8‐Ubx monomers to those of EGFP monomers. Purified s8:LOO8‐Ubx soluble monomers fluoresced green, and the excitation and emission spectra of s8:LOO8‐Ubx monomers matched those of EGFP and of EGFP‐Ubx monomers (Figure 3). EGFP is very stable and is known to retain activity in Ubx monomers and fibers (Z. Huang, Salim, et al., 2011; Nayak, et al., 2011). Additionally, the excitation and emission spectra of fibers composed of s8:LOO8‐Ubx matched those of fibers composed of EGFP‐Ubx (Figure 3, solid lines).

Thus, immobilization in Ubx materials did not appear to alter the function of LOO8 relative to its more stable counterpart, EGFP. Interestingly, the fluorescence emission spectra of both s8:LOO8‐Ubx and EGFP‐Ubx in fiber form are red‐shifted relative to the corresponding monomers (emission maximum shifts from 510 to 515 nm). Because both EGFP and LOO8 are affected, this red shift is not potentiated by the amino acid sequence alterations involved in creating LOO8. A likely explanation is that fiber formation places charged amino acids near the fluorescent proteins. A charged environment decreases the energy of the highly polarized excited state of the chromophore, red‐shifting the emission spectrum (Vivian & Callis, 2001). The largest density of charged residues in LOO8‐Ubx is in the Ubx homeodomain, in which 17 of the 60 amino acids are lysine or arginine. Most of these positively charged amino acids are located in the disordered N‐terminal arm and helix three of the homeodomain. Thus, negatively charged residues on the surface of LOO8 may form ionic bonds with the positively charged residues in the Ubx homeodomain, leading to a red shift in fluorescence by the chromophore. This red‐shift phenomenon has previously been observed for dityrosine bonds in Ubx, in which the blue fluorescence emission peak is shifted from the normal range of 410–430 to 438 nm (Howell et al., 2015). These data suggest that the ability of LOO8 to fluoresce is not impaired by fusion to Ubx or by Ubx polymerization.

### 2.2 Eliminating the background fluorescence in LOO8 biosensors

To optimize these sensors for use, we must first establish conditions that produce as low a baseline signal as possible. The left‐out peptide s8 is co‐expressed with LOO8‐Ubx to reconstitute protein folding and allow chromophore maturation. Naturally, priming s8 must be removed from the fibers by unfolding/refolding before the fibers can be used as a biosensor. This process produces apo·LOO8‐Ubx. There are many possible methods to unfold LOO8 and remove the priming s8. An ideal method would remove all of the s8 peptide, creating the lowest possible baseline signal, without compromising the LOO8 protein and, thus, reducing the maximum obtainable signal.

Our initial test used a buffer at pH 2 for 5 s to loosen s8:LOO8 and allow the priming s8 peptide to escape the fiber. This low pH was previously used to unfold LOO7 monomers (Y.‐M. Huang & Bystroff, 2009). When fibers composed of 100% LOO8‐Ubx were exposed to this buffer, they immediately lost fluorescence. However, after washing and returning the fibers to neutral pH, a substantial amount of fluorescence remained (Figure 5a). These data suggest that either (i) the LOO_GFPs are still oligomerizing, (ii) priming s8 is not being released from the LOO8 protein, or (iii) that priming s8 is not able to diffuse out of the materials in the time allowed and was rebinding to refolded LOO8. We also noted that peptide would not rebind previously denatured fibers, a problem addressed in the section on “Enhancing the signal in LOO8 biosensors” below.

**FIGURE 4:**
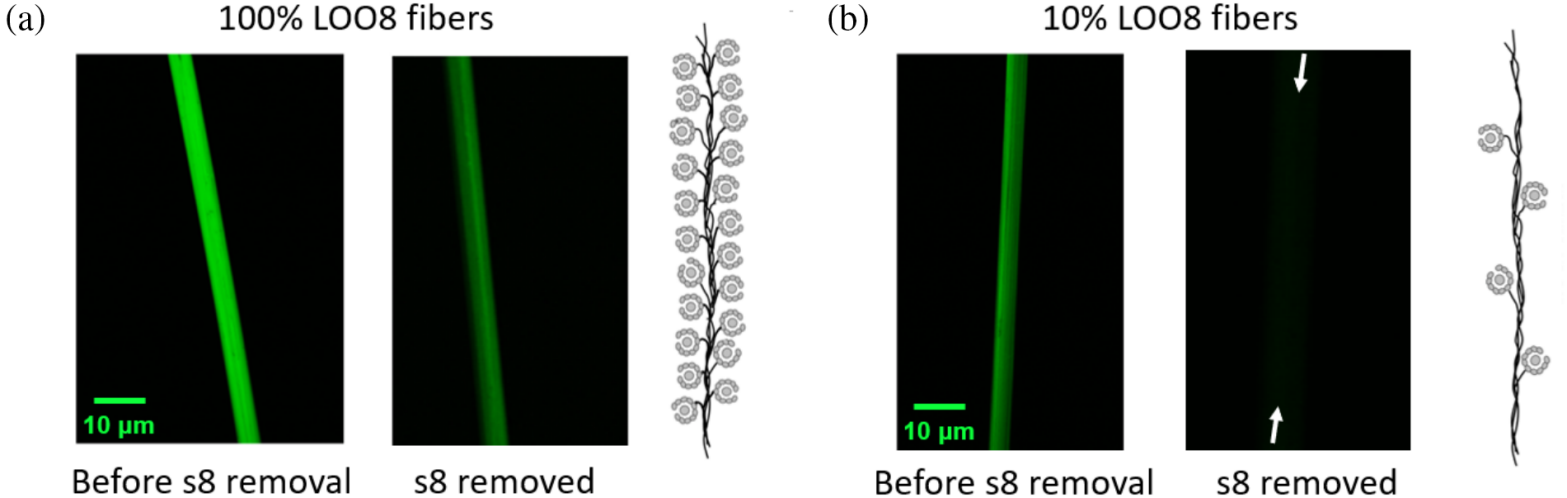
Concentration dependence of fiber fluorescence. (a) Confocal microscopy images of fibers composed of 100% LOO8‐Ubx before and after priming s8 removal by washing at pH 2 for 10 min followed by return to pH 8 buffer, at which point 45% of the fluorescence signal remains. (b) To prevent oligomerization and reduce background signal, many of the experiments in this paper used a mixture of 10% LOO8‐Ubx and 90% Ubx. These fibers still fluoresce green, albeit with less intensity, before s8 removal. In this formulation, little fluorescence can be detected in the absence of s8. Arrows mark the position of the fiber. The remaining fluorescence is only 18%. Cartoons depicting 100% LOO8 incorporation versus 10% LOO8 incorporation are shown to the right of (a) and (b). LOO, leave‐one‐out; Ubx, Ultrabithorax.

We first addressed the potential for LOO8 oligomerization to contribute to the background fluorescence. In a previous study of soluble LOO7, the intensity of background fluorescence caused by apo·LOO7 oligomerization was so severe that it rivaled that of the holo·LOO7:s7 peptide complex (Y.‐M. Huang et al., 2015). A goal of this study is to determine whether immobilizing LOO8 in Ubx materials can prevent LOO8 oligomerization and the resulting increase in background fluorescence. For our initial studies, we used materials composed of 100% LOO8‐Ubx (Figure 4a). The extent of fluorescence in 100% LOO8‐Ubx fibers after removing priming s8 was significantly reduced compared to before s8 removal, suggesting LOO8‐Ubx fibers have less oligomerization‐associated fluorescence than previously observed for soluble LOO7. This effect could be due to using LOO8, which is more soluble, or to immobilizing LOO8 in Ubx materials. Despite this improvement, some fluorescence remained in apo·LOO8‐Ubx fibers indicating that oligomerization may still occur. To further reduce LOO8 oligomerization, we also created materials composed of 10% LOO8‐Ubx and 90% unmodified Ubx (Figure 4b). We chose 10% LOO8 to try to prevent LOO8‐LOO8 interactions within the material. While we do not know the packing geometry for Ubx materials, we reasoned that common packing geometries have 10 or fewer nearest neighbors: cubic packing geometry would result in 6 nearest neighbors, body‐centered cubic packing geometry has 8 nearest neighbors, and a tetrahedral packing geometry would result in 10 nearest neighbors. Consequently, using 10% LOO8‐Ubx in materials gives us the largest possible signal while also minimizing the chances of contact between nearby LOO8 moieties. Quantitatively reducing the concentration of the cargo LOO8 protein is possible in LOO8‐Ubx materials because GFP‐Ubx and plain Ubx self‐assemble at the same rate (Tsai et al., 2015). Thus, mixing soluble GFP‐Ubx and plain Ubx monomers in a specific ratio will produce fibers composed of these proteins in the same ratio. Although reducing the ratio of LOO8 does weaken the signal, the concentration of LOO8 in the materials is roughly 25 mM, which is still 1000‐fold higher than the solubility limit of GFP in water (Tsai et al., 2015). Thus, the signal is still significantly elevated compared to LOO8 in solution. As expected, the 10:90 mixture produced morphologically normal fibers (Figure 4b). In theory, at this ratio we expect that the instances of LOO8 oligomerization will be reduced, thus reducing background fluorescence. In practice, these fibers ranged from displaying no background fluorescence to exhibiting some residual fluorescence after priming s8 removal. To achieve consistent results, we must also eliminate other possible sources of background fluorescence.

**FIGURE 5:**
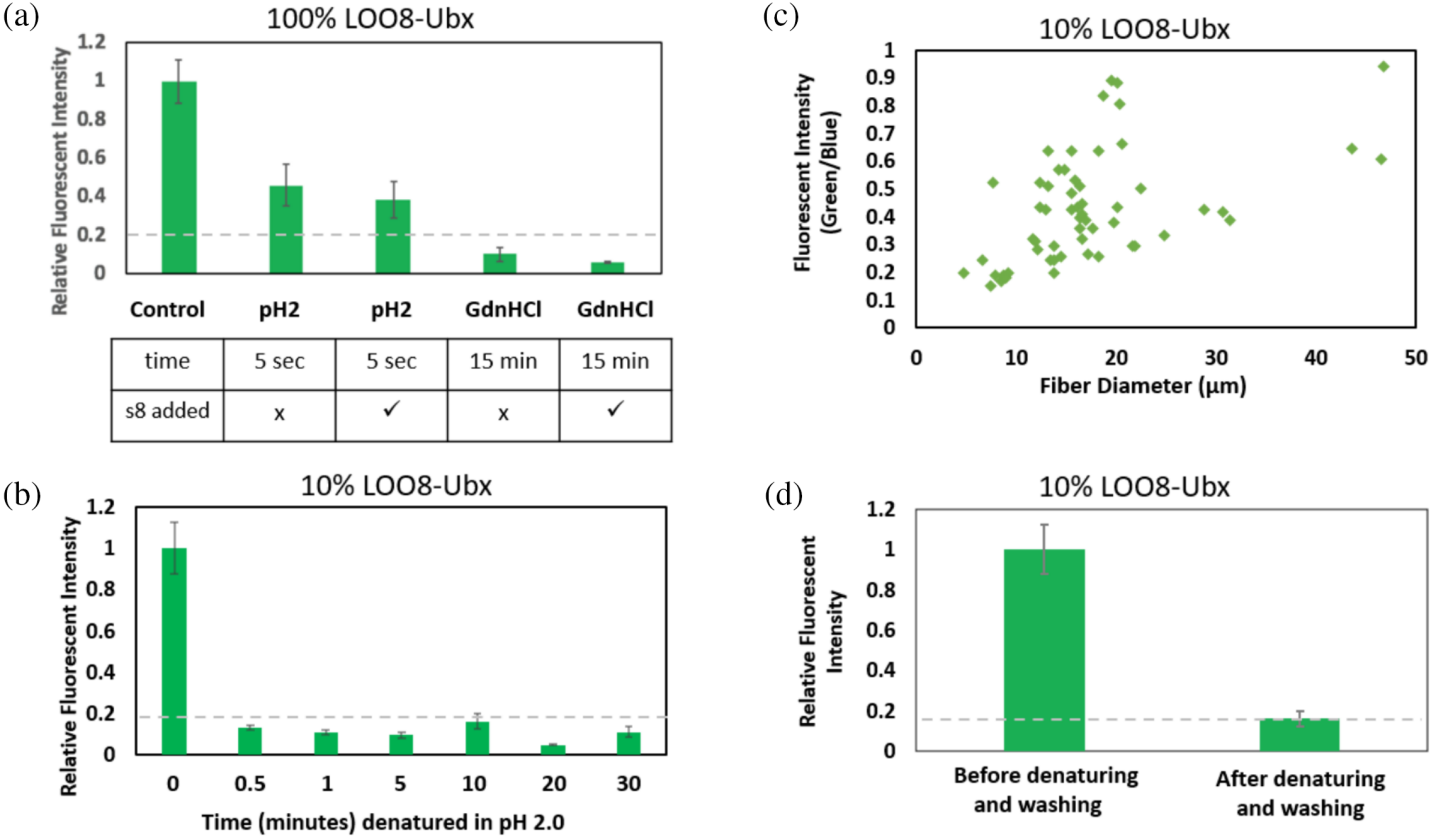
Conditions for removal of the priming peptide. The relative fluorescent intensity normalizes the intensity of green fluorescence against (1) the intensity of blue dityrosine fluorescence, which reports on the density of protein in the material, and (2) by diameter (except c), which reflects the amount of material present. The relative fluorescent intensity of control samples (LOO8‐Ubx still harboring the priming peptide) was set to 1. Dashed lines indicate the ambient signal in the absence of sample. (a) Fluorescent intensity of fibers composed of 100% LOO8‐Ubx is shown. Fibers were either denatured for 5 s in a pH 2 glycine buffer or for 15‐min in 4.5 M GdnHCl and washed in PBS. Finally, synthetic s8 was added as indicated to assess rebinding. (b) Relative fluorescent intensity of washed fibers after being denatured for varying amounts of time. No significant difference in background was measured between 30 s and 30 min. (c) Scatter plot of fiber diameter versus residual fluorescence (green/blue) for each fiber >50 μm. Although the relative fluorescent intensity is comparable to baseline for all samples, thicker fibers (>40 μm) have higher recovery of fluorescence in the absence of synthetic s8 after 10 min of denaturation than thinner fibers (<10 μm), indicating that some residual priming s8 remains in the thicker fibers. (d) Relative fluorescent intensity of 10% LOO8‐Ubx fibers 10–20 μm in diameter before and after denaturing in pH 2 buffer for 10 min and washing in PBS. Error bars represent standard error, *n* ≥ 3. By (1) starting with a more soluble LOO8 monomer, (2) reducing the concentration of LOO8 proteins in Ubx materials, and (3) optimizing the denaturing buffer, incubation time, and fiber diameter, we can reliably eliminate the background fluorescence caused by oligomerization (Figure 5d). LOO, leave‐one‐out; PBS, phosphate‐buffered saline; Ubx, Ultrabithorax.

A second possible source of background fluorescence is failure of the priming peptide to be released from LOO8. For each fiber measurement, we accounted for differences in the size of the sample by normalizing the intensity of green fluorescence against the intensity of blue dityrosine fluorescence, which reports on the density of protein in the material, and by diameter, which measures the amount of material present. Since incubating the fibers for 5 s at pH 2 failed to remove all background fluorescence, we increased both the strength of the denaturant and the incubation time. Exposure of the fibers for 15 min to 4.5 M GdnHCl, which acts as a stronger denaturant than acid for many proteins (Balacescu et al., 2020), should give ample opportunity for priming s8 to be released from LOO8. However, this treatment appears to damage the LOO8 protein and prevent rebinding, as subsequent addition of synthetic s8 failed to recover any signal (Figure 5a). Previous work suggested that proline isomerization or other slow changes in the unfolded state of GFP could lead to a “trapped” nonfluorescent state (Rosenman et al., 2014). Since we do not need to completely denature LOO8, only loosen its structure enough to dislodge the priming s8 peptide, we reverted back to the gentler acid exposure technique in the hopes of preventing conformational drift, but increased the denaturation time to provide more opportunity for s8 to dissociate from LOO8. By selecting an appropriate protein concentration and incubation time, most fibers (>90%) have diameters within this range. Larger and smaller fibers were discarded.

For all denaturation times tested (30 s to 30 min), after the subsequent wash steps no residual fluorescence was detected above background levels (Figure 5b). If conformational drift and/or proline isomerization was occurring during acid denaturation, then shorter incubation times would favor refolding (Andrews et al., 2009; Y.‐M. Huang & Bystroff, 2009). Removal of fluorescence is achieved quickly and remains the same for all time points. To balance the need to minimize conformational drift (short) denaturation times, while allowing sufficient time for the priming s8 to reliably dissociate from LOO8 and diffuse out of the fibers, we adopted a standard 10‐min denaturation time for all subsequent experiments. This approach also allowed synthetic s8 to rebind LOO8 and generate a fluorescent signal (described in Enhancing the Signal in LOO8 Biosensors, below).

The third possible source of background fluorescence is that priming s8 may fail to escape materials, and rebind after the denaturation step. Larger diameter fibers will require more time for priming s8 to diffuse out of the fibers and for analyte to diffuse into the fibers. Thus, diameter may affect measurement of both the observed background and the reconstituted fluorescence. We tested whether the fluorescence remaining after denaturing and washing is dependent on fiber diameter (Figure 5c). If no peptide is present, the green fluorescence from the (inactive) sensor, divided by the blue fluorescence used to account for fiber density should be zero. Because scattering of ambient light creates some background signal (gray dashed lines in Figure 5), and fibers block the scattered light, the signal in the absence of peptide is less than the background signal. If the peptide were completely absent, then larger fibers would block more ambient signal, and the measured green/blue fluorescence would slope down with increasing fiber diameter. Instead, we observed an upward slope, which we suspect is due to binding by peptide that has not escaped the fiber. Very thin fibers (<5 μm) are prone to breakage and are difficult to manipulate; thus, we chose fibers with diameters in the 10–20 μm range to minimize both breakage and trapping of the priming peptide within fibers in further experiments.

### 2.3 Enhancing the signal in LOO8 biosensors

The other approach to increase the signal‐to‐noise ratio of a biosensor is to maximize the detectable signal. In soluble LOO7 biosensors, the holo‐LOO7 peptide complex was only 40% as fluorescent as its parent protein, GFP OPT. The intensity of GFP fluorescence is inversely related to the dynamics of its chromophore, which, in turn, is dependent on GFP stability (Zhang et al., 2014). Not surprisingly, removing a GFP strand and replacing it with a separate peptide destabilizes the protein, accounting for the reduction in fluorescence in the holo‐LOO7 peptide complex. Consequently, there is plenty of room to improve the amount of signal generated by LOO_GFP proteins.

**FIGURE 6:**
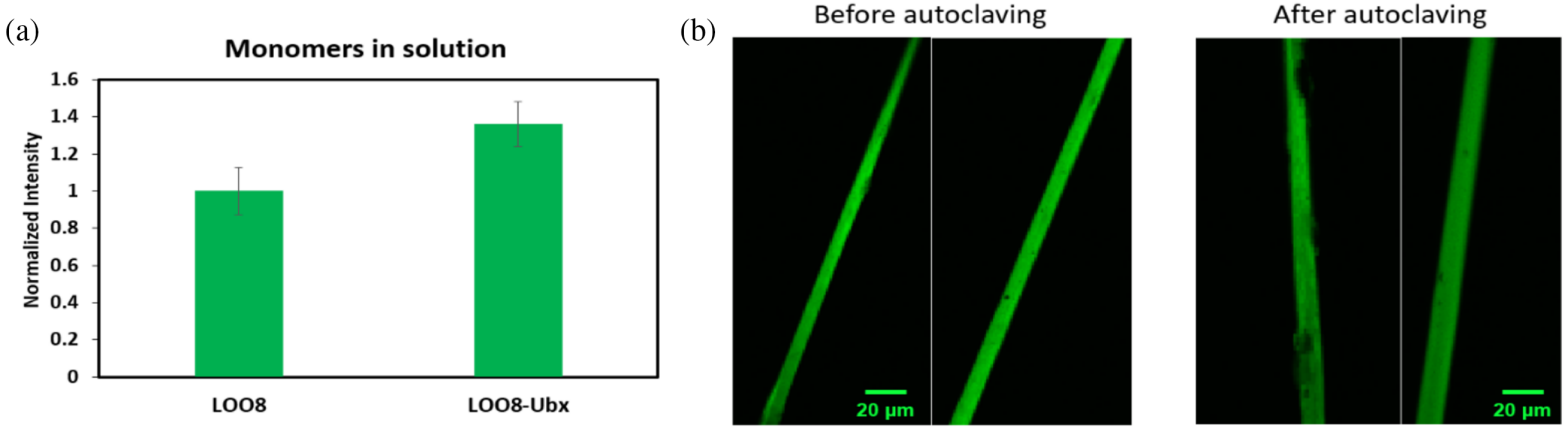
LOO8 is stabilized by fusion to Ubx. (a) Fluorescent intensity of LOO8 and LOO8‐Ubx monomers normalized by protein concentration. LOO8‐Ubx is 1.4 times as fluorescent as LOO8, a significant improvement (*p* < 0.05, *n* ≥ 3). Error bars represent standard error. (b) Representative confocal microscopy images of fibers before (two images on left) and after (two images on right) being autoclaved for 20 min. The first image on the right shows some pitting in the fiber caused by autoclaving, but most of the fluorescence is retained. LOO, leave‐one‐out; Ubx, Ultrabithorax.

Both fusion to Ubx and materials assembly have the potential to impact LOO_GFP stability, and thus LOO_GFP fluorescence. To determine if fusion to Ubx alters LOO8 fluorescence, we compared the relative fluorescence intensity of s8:LOO8 to s8:LOO8‐Ubx monomers, both at 5 nM. Surprisingly, Ubx fusion does enhance LOO8 fluorescence: LOO8‐Ubx was 1.4‐fold as fluorescent as unfused LOO8 (Figure 6a). Given that LOO8 is predicted to have a net negative charge, it is possible that intramolecular charge–charge interactions between the fluorescent proteins and the positively charged Ubx homeodomain enhance LOO8 stability, and hence the fluorescence, of these monomeric fusions. This hypothesis is supported by the prior observation of a higher breaking strain in EGFP‐Ubx fibers (Tsai et al., 2015), which was attributed to intermolecular EGFP:Ubx interactions, and in the red shift of the LOO8‐Ubx fiber emission spectrum (Figure 3). Another possibility is that the electrostatic interactions with Ubx stabilize the excited state of the chromophore, a state in which there is increased charge separation relative to the ground state. When excited by 490 nm light, such stabilization could increase the concentration of the excited state of LOO8‐Ubx relative to monomeric LOO8, increasing quantum yield.

We have previously demonstrated that Ubx materials can stabilize other proteins to a surprising extent. Indeed, EGFP‐Ubx fibers can be autoclaved or stored at room temperature for >9 years and remain intact and green fluorescent (Tsai et al., 2015). Stabilization of cargo proteins fused to protein‐based materials has been observed for other materials as well and is attributed to confined geometry effects. We repeated these experiments with s8:LOO8‐Ubx and found that autoclaved LOO8‐Ubx fibers still retained significant fluorescence (Figure 6b). Fibers soaked in 100% ethanol for 30 min also remained fluorescent (data not shown). These fluorescent data from monomers and fibers suggest a substantial increase in protein stability, which may further elevate the signal provided by LOO8 when incorporated into Ubx materials. In the long term, this extreme resistance to high temperature may allow biosensor distribution and use without a cold storage chain.

### 2.4 Optimization of conditions to reconstitute fluorescence

**FIGURE 7:**
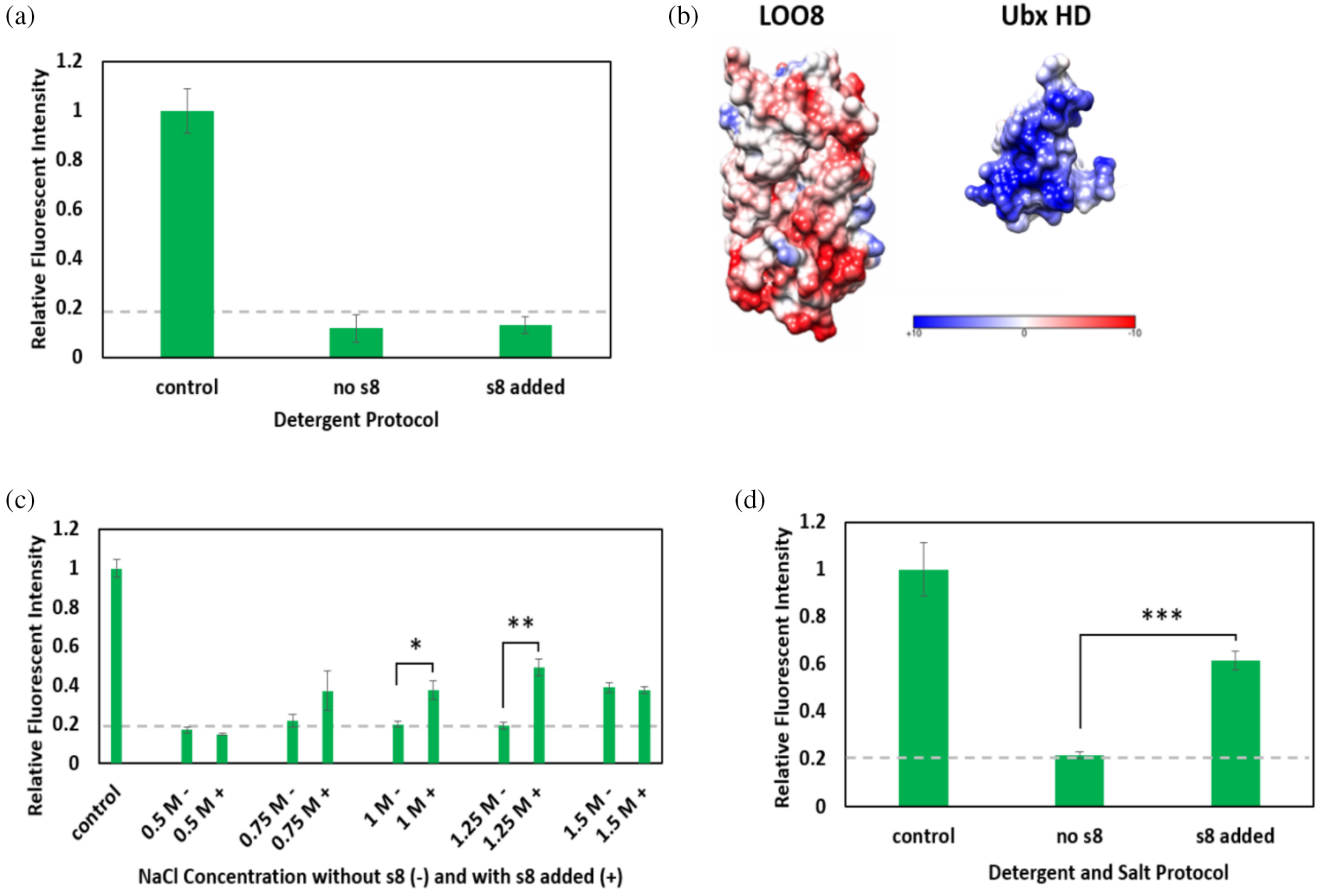
Optimization of peptide rebinding conditions. Fluorescent intensity data were collected from confocal microscopy images of treated and untreated fibers and normalized against the blue fluorescence and diameter of each fiber to account for sample volume and Ubx packing density. Three measurements from each fiber image were taken to get an average intensity value for each sample. The dashed line indicates the ambient signal in the absence of sample. (a) Fluorescent intensity of fibers relative to untreated fiber intensity (control) is shown with and without synthetic s8 added after denaturing with pH 2 buffer containing 0.1% SDS and washing with buffer containing 0.1% CHAPS, pH 8. None of the samples showed recovery of fluorescence above the background of the microscope (gray dashed line). (b) Cartoon of LOO8 (left, PDBID: 2b3p) showing the negative surface charge (red) around the binding pocket for s8 and the positive surface charge (blue) on both helix 3 and the disordered N‐terminal arm of the Ubx homeodomain (right, PDBID 1B8I). Images are not to scale. (c) Fluorescent intensity of fibers relative to untreated controls is shown with (+) and without (−) synthetic s8 added after denaturing and washing with different concentrations of NaCl. Samples denatured and washed in buffers containing 1 M NaCl and 1.25 M NaCl showed a significant increase in fluorescent intensity above the background. (d) Fluorescent intensity of fibers relative to untreated controls is shown with and without synthetic s8 added to fibers denatured with 1.25 M NaCl and 0.1% SDS and washed with 1.25 M NaCl and 0.1% CHAPS. These conditions are summarized in Figure 8. Error bars represent standard error. Significance determined by two‐sample *t*‐test for equal means, *p < 0.05, **p < 0.001, ***p < 0.000001, *n* ≥ 3. LOO, leave‐one‐out; Ubx, Ultrabithorax.

The next challenge was to identify conditions in which synthetic s8 reliably rebinds the apo·LOO8‐Ubx fiber, displaying the fluorescent signal. Our initial experiment using phosphate‐buffered saline (PBS) as the rebinding buffer did not reveal any synthetic s8 rebinding above background levels (Figure 5a). In previous LOO7_GFP studies, the s7 peptide was removed while LOO7_GFP was immobilized on a Ni‐NTA agarose column. The pH was lowered to 2.0, then SDS was added to remove s7 prior to washing with CHAPS, a weak detergent, at pH 8.0. This method was found to recover more of the fluorescence than a simple pH jump or by only adding and removing GdnHCl (Y.‐M. Huang et al., 2015; Y.‐M. Huang & Bystroff, 2009). However, when we included sodium dodecyl sulfate (SDS) in the denaturing step and CHAPS in the washes, we did not observe any synthetic s8 rebinding (Figure 7a). An important clue to the lack of s8 rebinding was provided by a prior study in which ionic interactions between Ubx (predicted net charge +9) and EGFP (predicted net charge −2) were proposed to be the source of the increased strength of EGFP‐Ubx fibers relative to fibers composed of only Ubx (Tsai et al., 2015). Many of the negatively charged residues in apo·LOO8 surround the gap created when priming s8 is removed (Figure 7b). We hypothesized that in the absence of s8, the negatively charged face of apo·LOO8 binds Ubx, which then physically blocks synthetic s8 rebinding. Indeed, the red shift of the LOO8 emission spectrum in fibers versus monomers (Figure 3) and stabilization of LOO8‐Ubx monomers (Figure 6) further suggests such interactions may be occurring. If true, increasing the salt concentration should reduce this effect. We repeated synthetic s8 binding experiments with different concentrations of NaCl in both the denaturation and wash buffers to screen any ionic interactions between LOO8 and Ubx and allow synthetic s8 to rebind and reconstitute fluorescence. For each NaCl concentration, we compared samples in which synthetic s8 was added in the final step after washing, to samples in which s8 was omitted, to assess the levels of background fluorescence. Below 0.75 M NaCl, adding synthetic s8 did not recover significant fluorescence above background, suggesting this concentration was not sufficient to screen the charge interactions. At 1.5 M NaCl, the background is high and indistinguishable from the fluorescence recovered by adding synthetic s8. This very high salt concentration appears to inhibit escape of the priming s8 peptide from the fibers during the denaturation and washing steps. The retention of the peptide could be caused by several mechanisms, including contraction of the materials in response to high salt, as previously observed for silk (Kamada et al., 2023), or a reduction in the solubility of the LOO8 protein at high salt concentrations (Ishii et al., 2007). However, with 1 M and 1.25 M NaCl concentrations, we observe a significant increase in fluorescence over the background when synthetic s8 is added (Figure 7c).

After successfully using salt to screen charge interactions between LOO8 and Ubx, we revisited the previously described protocol of adding detergents to the denaturation and wash steps to determine if recovery of fluorescence could be further improved. SDS was included in the denaturation buffer, and the salt concentration in the first wash buffer was lowered to ensure the detergent would interact with the fibers. Subsequent wash steps reverted to high salt to facilitate peptide removal and included 3‐((3‐cholamidopropyl) dimethylammonio)‐1‐propanesulfonate (CHAPS), a weaker detergent, to help remove the SDS. When we used 1.25 M NaCl in combination with the detergent, we observed greater recovery of fluorescence (Figure 7d) than when using 1.25 M NaCl alone (Figure 7c). We hypothesize that the addition of a detergent loosens LOO8 structure and facilitates diffusion of priming s8 out of and into the fibers, increasing the efficiency of the washes. Use of these optimized conditions confirms that the LOO8 biosensors can function properly when immobilized within protein fibers.

Covalently immobilizing biosensors can not only facilitate their use but also improve biosensor function. Fusion of the LOO8 biosensor to Ubx increases the fluorescent signal of LOO8‐Ubx monomers over LOO8 monomers by 1.4‐fold. Immobilization in Ubx materials also prevents LOO8 oligomerization, thus removing the associated background fluorescence. Immobilization of LOO8 in materials also stabilizes the sensor, allowing most of the fluorescence to be retained after autoclaving for 20 min or soaking in ethanol for 30 min. This extreme stabilization greatly increases the viable lifetime of the sensor, potentially enabling the biosensor to be deployed without maintaining a cold chain for storage.

**FIGURE 8:**
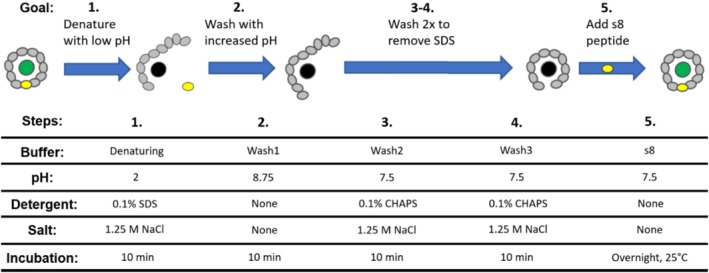
Optimized conditions for the LOO8‐Ubx biosensor. The optimal conditions for removing fluorescence consider the charge distribution of the proteins and size of the materials. For 10% LOO8‐Ubx materials of 10–20 μm diameter, we use a pH 2.0 denaturing buffer with 1.25 M NaCl and 0.1% SDS (Step 1). The first wash with PBS raises the pH back to 8.0 and does not add any additional salt or detergent (Step 2). The second and third washes shield charge interactions with 1.25 M NaCl and use CHAPS to remove the SDS (Steps 3 and 4). Finally, synthetic s8 is added to recover fluorescence (step 5). LOO, leave‐one‐out; PBS, phosphate‐buffered saline; Ubx, Ultrabithorax.

Interactions between materials and sensors can both benefit and/or harm the biosensor and will depend on the nature of both entities. For this work, expression tests of LOO7‐, LOO8‐, and LOO9‐Ubx found that LOO8‐Ubx had the best expression in *Escherichia coli* (data not shown). Therefore, the production of Ubx fusion monomers is sensitive to the protein being appended, even when comparing different LOO proteins, which have nearly the same composition as each other and their parent protein. Immobilization also required optimization of other parameters. We expected we would have to alter incubation and wash times to allow diffusion of the target peptide in and out of the Ubx fibers. Because Ubx and LOO8‐Ubx self‐assemble equally well, we also had the opportunity to adjust the percent of fused monomers in the materials, and thus prevent LOO8 aggregation. Finally, unforeseen interactions between the apo·LOO8 biosensor and Ubx hampered synthetic s8 rebinding. Although buffer conditions had to be optimized for our assays, we were able to develop a protocol to address this issue by using elevated salt concentrations and including detergents in the wash steps (Figure 8). The net result is a sensor that reproducibly gives a significant readout signal above the background, a substantial improvement over the function of the LOO monomers. Thus, immobilizing LOO8 in Ubx materials successfully improved the function of the LOO8 biosensor, increasing the signal‐to‐noise by approximately fourfold (Table 1). These results also demonstrate the potential for both protein fusion and materials assembly to alter the function of the cargo protein. Consequently, there is an urgent need to develop an array of materials‐forming proteins to successfully overcome different potentially detrimental protein–protein interactions and provide opportunities for protein–material interactions that enhance function.

**TABLE 1:** Improving the signal‐to‐noise ratio of LOO8‐Ubx fibers by altering assay conditions.

Condition	Description	S/N	Fold improvement versus prior step	Overall fold improvement
1	100% fiber, long denaturation	0.9		
2	10% fiber, short denaturation	1.2	1.3×	1.3×
3	Condition 2 + Salt	3.0	2.5×	3.3×
4	Condition 2 + Salt and Detergent	3.6	1.2×	4×

This report presents the first‐ever immobilization of a protein complementation assay‐based biosensor in protein materials. As an immobilization technique, gene fusion has many advantages. Because both the material and biosensor are produced as a single molecule, the cost and time required for multiple preparations and tethering the biosensor are saved. Unlike prior noncovalent attachment of LOO_GFP biosensors to resin, the covalent attachment provided by the gene fusion technique is expected to substantially reduce sensor leaching. Finally, as noted herein, immobilization in the materials can stabilize the biosensor, increase the maximum signal, and reduce background fluorescence.

Despite the many advantages of gene fusion, this technique is most frequently used only for short peptides and a limited number of small, monomeric, single‐domain proteins. A small number of homodimeric and homotetrameric proteins have also been successfully incorporated into protein‐based materials via gene fusion (Hino et al., 2006; Thatikonda et al., 2018; Tsai et al., 2015). To our knowledge, this paper reports the first‐ever functional heterodimer (s8 and LOO8) incorporated into protein‐based materials. Inclusion of the heterodimer is achieved via two methods. In the first, LOO8‐Ubx is co‐expressed with the s8 peptide to create a functional fluorescent protein capable of self‐assembling into materials. In the second approach, materials composed of apo·LOO8 bind the free s8 peptide to reconstitute the heterodimeric structure post‐assembly and allow LOO8 to fluoresce. The ability to incorporate heterodimers with a desired biochemical function into materials composed of a self‐assembling protein via gene fusion greatly expands the potential of this technique, since many functional proteins that are desirable to incorporate into materials, including numerous enzymes and growth factors, are heterodimers.

## 3 CONCLUSIONS

In this study, we created peptide‐sensing materials by embedding, for the first time, a protein complementation assay in protein‐based materials via gene fusion. Importantly, the materials are assembled while bound to the target peptide, which also makes this paper the first report of protein materials incorporating a heterodimeric protein via gene fusion. Immobilization in Ubx materials substantially increased the fluorescent signal from LOO8 and decreased background fluorescence by preventing LOO8 oligomerization. However, we also discovered that noncovalent interactions between the LOO8 sensor and the self‐assembling Ubx protein could impede peptide release and rebinding. These issues could be addressed by altering the composition of the buffers used during the assay to mitigate LOO8–Ubx interactions. These results provide both a cautionary tale and a potential solution for other scientists working with protein‐based materials.

## 4 MATERIALS AND METHODS

### 4.1 Construction of plasmids

Sequences of all proteins are provided in the Supporting Information (Table S1). The pCDFDuet‐1‐LOO8‐GFP plasmid (Y. M. Huang, Nayak, et al., 2011) was used as the parent vector to co‐express LOO8‐Ubx and its left‐out peptide (s8). DNAs encoding the LOO8‐Ubx fusion protein and s8 were inserted at sites termed “MCS1” and “MCS2,” respectively. Ubx splicing isoform Ia (GenBank AAN13718.1) was inserted between the BamHI and EcoRI sites in the MCS1 site. MCS2 contains the left‐out peptide “NGIKANFTVRHNV,” termed “priming s8,” fused C‐terminally to the carrier protein Ssp‐DnaB mini‐intein by insertion into the BglII and EcoRV sites, as previously described (Z. Huang, Salim, et al., 2011). Thus, s8 is produced as a fusion protein and subsequently cleaved to create free s8 peptide.

The pET19b‐UbxIa and pET19b‐EGFP‐UbxIa plasmids were also used. EGFP is a variant of GFP that has a greater fluorescent intensity and higher folding efficiency than wild‐type GFP (Cormack et al., 1996). As previously described, these constructs contain the Ubx mRNA splicing isoform Ia (GenBank AAN13718.1) inserted between the NdeI and BamHI sites of the pET19b vector (Novagen) (Tsai et al., 2015). pET19b‐EGFP‐Ubx1a also includes EGFP inserted in the NdeI site between the N‐terminal His‐tag and Ubx. EGFP and Ubx are separated by a short linker sequence “GSGSHMGSGS” to allow proper folding of both proteins.

### 4.2 Protein expression, purification, and materials production

Protocols were used as established for expression and purification of Ubx and EGFP‐Ubx protein fusions into materials (Greer et al., 2009; Tsai et al., 2015). To express LOO8_GFP‐Ubx and its cognate peptide, the pCDFDuet‐1‐LOO8_GFP plasmid was transformed into Rosetta (DE3) pLysS cells (Novagen). Single colonies were used to inoculate overnight liquid cultures, and 10 mL of the overnight culture was used to inoculate a 1 L LB culture. Both cultures contained 0.03 mg/L chloramphenicol and 0.03 mg/L streptomycin. Protein expression was induced at mid‐log phase (OD_600_ = 0.6) with 1 × 10^−3^ M isopropyl‐β‐d‐1‐thiogalactopyranoside for 5 h; then cells were harvested by centrifugation at 7000*g* for 10 min at 4°C and stored at −20°C. Frozen cell pellets corresponding to 2 L of cell culture were lysed according to established protocols (Greer et al., 2009, Tsai et al., 2015) in a lysis buffer containing 50 mM NaH_2_PO_4_, 500 mM NaCl, 5% glucose, 20 mM imidazole, 1.5 mM PMSF, 5 mM DTT, 0.2 mg/mL lysozyme, two tablets of Complete Proteinase Inhibitor Mixture (Roche), and 0.04 mg/mL DNase I at pH 8.75. Cell debris was removed by centrifugation at 18,000*g* for 30 min at 4°C. Protein was purified from the clarified cell lysate using 5 mL HisPur™ Ni‐NTA Superflow Agarose slurry (Thermo Fisher Scientific), which was poured into a column and pre‐equilibrated with 100 mL of wash buffer (50 mM NaH_2_PO_4_, 500 mM NaCl, and 5% glucose at pH 8.0). Bound resin was washed with 50 mL of wash buffer and 50 mL of wash buffer containing 20 mM imidazole. Protein was eluted using a buffer containing 50 mM NaH_2_PO_4_, 500 mM NaCl, 5% glucose, and 300 mM imidazole at pH 7.0. Proteins were >90% pure as evaluated by gel electrophoresis (Figure S1). The concentration of purified protein in samples was determined using the BioRad protein assay (BioRad). Fibers were pulled from films produced in a “buffer reservoir” (Mendes et al., 2018) with 1–2 mg of protein per reservoir, which contained 250 mL of the wash buffer described above. After a 10‐min incubation, films were compressed by drawing a plastic bar across the surface of the buffer; then fibers were pulled from the films and wrapped around 5‐mm sterile plastic inoculation loops (hereafter, “loops”). Fibers on loops were allowed to air‐dry and stored upright in air until use, typically within 1–3 days. To prevent LOO8 oligomerization, all fibers used for priming, s8 removal, and rebinding experiments were composed of 10% LOO8‐Ubx and 90% Ubx, unless otherwise noted. Creating fibers with defined mixtures of proteins is possible because EGFP‐Ubx and plain Ubx self‐assemble equally well (Tsai et al., 2015).

### 4.3 Spectroscopic measurements

Fluorescence spectroscopy was performed using a JASCO‐815 fluorometer and a 10‐mm pathlength quartz cuvette. Proteins were diluted into wash buffer, described above, to a concentration of 5 nM. Excitation spectra, read at 490 nm, and emission spectra, read at 510 nm, were monitored at 23°C using a bandwidth of 1 nm for samples in solution, and the maximum peak intensity was defined as 1. For fluorescent intensity measurements of LOO8 and LOO8‐Ubx monomers, samples were diluted in water to 100 nM and excited at 490 nm. The emission spectra were collected with a slit width of 10 nm at 23°C, and the maximum peak intensity was defined as 1.

### 4.4 Denaturing assay

To determine the effect of denaturing washes on the removal of bound priming s8, loops supporting 4–6100% LOO8‐Ubx fibers were placed in a 24‐well plate and incubated in 1 mL of 0.1 M glycine‐HCl buffer, pH 2.0 for 5 s or 4.5 M GdnHCl for 15 min. Fibers were rinsed by quickly submerging the loops in 2 different wells, each containing 1 mL of PBS (20 mM NaH_2_PO_4_, 80 mM Na_2_HPO_4_, 500 mM NaCl, pH 8.0), then submerging the loop in fresh PBS for 10 min. After this final wash, fibers were imaged as described below.

### 4.5 Denaturing wash time assay

To test the time required to remove bound priming s8, loops supporting 4–6 10% LOO8‐Ubx fibers were placed in a 24‐well plate and incubated in 1 mL of 0.1 M glycine‐HCl buffer, pH 2.0 for 0.5, 1, 5, 10, 20, or 30 min. Fibers were rinsed by successively placing the loops in 3 different wells, each containing 1 mL of PBS for 10 min each. After the final wash, fibers were imaged as described below.

### 4.6 Fiber size assay

Loops with 10% LOO8‐Ubx fibers were placed in a 24‐well plate and denatured for 10 min to remove bound priming s8 with 1 mL of 0.1 M glycine‐HCl buffer, pH 2.0. Fibers were rinsed 3 times by incubating the loops in wells containing 1 mL of PBS for 10 min for each rinse. After the final wash, fibers were imaged as described below and sorted based on the fiber diameter, measured using confocal microscopy.

### 4.7 Detergent assay

Synthetic s8 peptide “NGIKANFTVRHNV” (1 mg, Genscript) was dissolved in 50 μL of dimethylformamide (Sigma) and diluted with PBS to a stock concentration of 0.12 mM, then aliquoted and stored at −80°C until use. Loops with 10% LOO8‐Ubx fibers were placed in a 24‐well plate and denatured for 10 min to remove bound priming s8 with 1 mL of denaturing buffer (50 mM Tris pH 2.0, 50 mM NaH_2_PO_4_, 300 mM NaCl, 0.1% SDS, 100 mM 2‐Mercaptoethanol (BME)). Fibers were rinsed by placing the loops in 1 mL of buffer A1 (50 mM Tris pH 8.75, 50 mM NaH_2_PO_4_, 300 mM NaCl) for 10 min, 1 mL of buffer A2 (50 mM Tris pH 7.5, 50 mM NaH_2_PO_4_, 300 mM NaCl, 10 mM imidazole, 0.1% CHAPS, 10 mM BME) for 10 min, and 1 mL of buffer A3 (50 mM Tris pH 7.5, 50 mM NaH_2_PO_4_, 300 mM NaCl, 20 mM imidazole, 0.1% CHAPS, 10 mM BME) for 10 min. Fibers were then incubated overnight either in PBS or 0.12 mM synthetic s8 (GenScript) diluted in PBS at 25°C to bind synthetic s8 before imaging as described below.

### 4.8 NaCl concentration assay

Loops with 10% LOO8‐Ubx fibers were placed in a 24‐well plate and denatured for 10 min to remove bound priming s8 with 1 mL of 0.1 M glycine‐HCl buffer, pH 2.0, containing elevated NaCl as indicated (0.5, 0.75, 1.25 or 1.5 M NaCl). Fibers were rinsed three times for 10 min each by placing the loops in different wells, each containing 1 mL PBS, supplemented with the same NaCl concentration used in the denaturing wash. Fibers were then incubated with either PBS or 0.12 mM synthetic s8 (GenScript) diluted in PBS and left overnight at 25°C to bind synthetic s8 before imaging as described below.

### 4.9 Detergent and NaCl assay

To remove bound priming s8, loops with 10% LOO8‐Ubx fibers were denatured for 10 min with 1 mL of high‐salt denaturing buffer (50 mM Tris pH 2.0, 50 mM NaH_2_PO_4_, 1.25 M NaCl, 0.1% SDS, 100 mM BME) in a 24‐well plate. Fibers were rinsed by submerging the loops in 1 mL of buffer A1 for 10 min, 1 mL of buffer A2++ (50 mM Tris pH 7.5, 50 mM NaH_2_PO_4_, 1.25 M NaCl, 10 mM imidazole, 0.1% CHAPS, 10 mM BME) for 10 min, and 1 mL of buffer A3++ (50 mM Tris pH 7.5, 50 mM NaH_2_PO_4_, 1.25 M NaCl, 20 mM imidazole, 0.1% CHAPS, 10 mM BME) for 10 min. Fibers were then placed either in PBS or 0.12 mM synthetic s8 (GenScript) diluted in PBS and left overnight at 25°C to bind synthetic s8 before imaging as described below.

### 4.10 Confocal images and fluorescent intensity measurements

For imaging, loops with fibers were transferred from the 24‐well plate to a glass slide or coverslip and kept hydrated with a drop of distilled water. Fibers were imaged using confocal microscopy on a Nikon Eclipse Ti A1R equipped with NIS Elements AR software to assess fiber diameter and for fluorescent intensity analysis. Images were captured using a 20× objective, and each experiment used untreated fibers as controls for fluorescent intensity. Fluorescent intensity of LOO8‐Ubx was normalized using fiber diameter and dityrosine fluorescence, measured in the 4′,6‐diamidino‐2‐phenylindole channel, which accounts for the amount of materials present (Howell et al., 2015). For each fiber image, background is defined as the detected light originating outside the resolution volume (i.e., a fiber) and signal is defined as the detected light originating within the resolution volume (Sandison & Webb, 1994). For measurements of nonfluorescent fibers, the fluorescent intensity, or signal, is below the background of the microscope due to a lack of light scattering within the fiber. Error bars represent standard error for all quantified data.

## AUTHOR CONTRIBUTIONS

**Rebecca M. Booth:** Writing – review and editing; investigation; methodology; writing – original draft. **Amanda Jons:** Writing – original draft; investigation; methodology; writing – review and editing. **Xue Gong:** Investigation; methodology; writing – review and editing. **Shounak Banerjee:** Conceptualization; methodology. **Britt Faulk:** Writing – review and editing; investigation. **Hays Rye:** Methodology; writing – review and editing; investigation. **Christopher Bystroff:** Conceptualization; methodology; writing – review and editing; funding acquisition; project administration. **Sarah E. Bondos:** Project administration; conceptualization; methodology; funding acquisition; writing – review and editing.

## CONFLICT OF INTEREST STATEMENT

SEB has research support from Bondwell Technologies, Inc., holds stock in Bondwell Technologies, Inc., and serves as its Chief Scientific Officer. The terms of this arrangement have been reviewed and approved by the Texas A&M University in accordance with its policy on objectivity in research. RMB and AJ are currently employed by Bondwell Technologies, but had no affiliation with the company during these studies.

## Supporting information


**Data S1.** Supporting Information.

## Data Availability

The data that support the findings of this study are available from the corresponding author upon reasonable request.

## References

[pro70119-bib-0001] Andrews BT , Roy M , Jennings PA . Chromophore packing leads to hysteresis in GFP. J Mol Biol. 2009;392(1):218–227. 19577576 10.1016/j.jmb.2009.06.072 PMC3566660

[pro70119-bib-0002] Balacescu L , Schrader TE , Radulescu A , Zolnierczuk P , Holderer O , Pasini S , et al. Transition between protein‐like and polymer‐like dynamic behavior: internal friction in unfolded apomyoglobin depends on denaturing conditions. Sci Rep. 2020;10(1):1570. 32005832 10.1038/s41598-020-57775-4 PMC6994677

[pro70119-bib-0003] Barondeau DP , Putnam CD , Kassmann CJ , Tainer JA , Getzoff ED . Mechanism and energetics of green fluorescent protein chromophore synthesis revealed by trapped intermediate structures. Proc Natl Acad Sci USA. 2003;100(21):12111–12116. 14523232 10.1073/pnas.2133463100 PMC218721

[pro70119-bib-0004] Bravaya KB , Grigorenko BL , Nemukhin AV , Krylov AI . Quantum chemistry behind bioimaging: insights from ab initio studies of fluorescent proteins and their chromophores. Acc Chem Res. 2011;45(2):265–275. 21882809 10.1021/ar2001556

[pro70119-bib-0005] Cabantous S , Terwilliger TC , Waldo GS . Protein tagging and detection using engineered self‐assembling fragments of green fluorescent protein. Nat Biotechnol. 2005;23(1):102–107. 15580262 10.1038/nbt1044

[pro70119-bib-0006] Calabrese A , Battistoni P , Ceylan S , Zeni L , Capo A , Varriale A , et al. An impedimetric biosensor for detection of volatile organic compounds in food. Biosensors Basel. 2023;13(3):341. 36979553 10.3390/bios13030341 PMC10046769

[pro70119-bib-0007] Cormack BP , Valdivia RH , Falkow S . FACS‐optimized mutants of the green fluorescent protein (GFP). Gene. 1996;173(1):33–38. 8707053 10.1016/0378-1119(95)00685-0

[pro70119-bib-0008] Crone DE , Huang Y‐M , Pitman DJ , Schenkelberg C , Fraser K , Macari S , et al. GFP‐based biosensors. State of the art in biosensors—general aspects. Rijeka: Intech; 2013. p. 3–36.

[pro70119-bib-0009] Diaspro A , Kroi S , Campanini B , Cannone F , Chirico G . Enhanced green fluorescent protein (GFP) fluorescence after polyelectrolyte caging. Opt Express. 2006;14(21):9815–9824. 19529373 10.1364/oe.14.009815

[pro70119-bib-0010] Dong T , Matos Pires NM , Yang Z , Jiang Z . Advances in electrochemical biosensors based on nanomaterials for protein biomarker detection in saliva. Adv Sci. 2023;10(6):2205429. 10.1002/advs.202205429 PMC9951322 36585368

[pro70119-bib-0011] Greer AM , Huang Z , Oriakhi A , Lu Y , Lou J , Matthews KS , et al. The drosophila transcription factor Ultrabithorax self‐assembles into protein‐based biomaterials with multiple morphologies. Biomacromolecules. 2009;10(4):829–837. 19296655 10.1021/bm801315v

[pro70119-bib-0012] Hassibi A , Vikalo H , Hajimiri A . On noise processes and limits of performance in biosensors. J Appl Phys. 2007;102(1):014909.

[pro70119-bib-0013] Hino R , Tomita M , Yoshizato K . The generation of germline transgenic silkworms for the production of biologically active recombinant fusion proteins of fibroin and human basic fibroblast growth factor. Biomaterials. 2006;27(33):5715–5724. 16905183 10.1016/j.biomaterials.2006.07.028

[pro70119-bib-0014] Howell DW , Tsai S‐P , Churion K , Patterson J , Abbey C , Atkinson JT , et al. Identification of multiple dityrosine bonds in materials composed of the drosophila protein Ultrabithorax. Adv Funct Mater. 2015;25(37):5988–5998. 28725173 10.1002/adfm.201502852 PMC5513195

[pro70119-bib-0015] Huang Y‐M , Banerjee S , Crone DE , Schenkelberg CD , Pitman DJ , Buck PM , et al. Toward computationally designed self‐reporting biosensors using leave‐one‐out green fluorescent protein. Biochemistry. 2015;54(40):6263–6273. 26397806 10.1021/acs.biochem.5b00786 PMC4939794

[pro70119-bib-0016] Huang Y‐M , Bystroff C . Complementation and reconstitution of fluorescence from circularly permuted and truncated green fluorescent protein. Biochemistry. 2009;48(5):29–940. 10.1021/bi802027g PMC4651016 19140681

[pro70119-bib-0017] Huang Y‐M , Nayak S , Bystroff C . Quantitative in vivo solubility and reconstitution of truncated circular permutants of green fluorescent protein. Protein Sci. 2011;20(11):1775–1780. 21910151 10.1002/pro.735 PMC3267941

[pro70119-bib-0018] Huang Z , Lu Y , Majithia R , Shah J , Meissner K , Matthews KS , et al. Size dictates mechanical properties for protein fibers self‐assembled by the *Drosophila* hox transcription factor Ultrabithorax. Biomacromolecules. 2010;11(12):3644–3651. 21047055 10.1021/bm1010992

[pro70119-bib-0019] Huang Z , Salim T , Brawley A , Patterson J , Matthews KS , Bondos SE . Functionalization and patterning of protein‐based materials using active Ultrabithorax chimeras. Adv Funct Mater. 2011;21(14):2633–2640.

[pro70119-bib-0020] Ishii M , Kunimura JS , Jeng HT , Penna TCV , Cholewa O . Evaluation of the pH‐ and thermal stability of the recombinant green fluorescent protein (GFP) in the presence of sodium chloride. Appl Biochem Biotechnol. 2007;137–140(1–12):555–571. 10.1007/s12010-007-9079-6 18478416

[pro70119-bib-0021] Jarczewska M , Malinowska E . The application of antibody–aptamer hybrid biosensors in clinical diagnostics and environmental analysis. Anal Methods. 2020;12(25):3183–3199. 32930180 10.1039/d0ay00678e

[pro70119-bib-0022] Kamada R , Miyazaki H , Janairo JIB , Chuman Y , Sakaguchi K . Bilayer hydrogel composed of elastin‐mimetic polypeptides as a bio‐actuator with bidirectional and reversible bending behaviors. Molecules. 2023;28(13):5274. 37446933 10.3390/molecules28135274 PMC10343461

[pro70119-bib-0023] Kodama Y , Hu C‐D . Bimolecular fluorescence complementation (BIFC): a 5‐year update and future perspectives. Biotechniques. 2012;53(5):285–298. 23148879 10.2144/000113943

[pro70119-bib-0024] Köker T , Fernandez A , Pinaud F . Characterization of split fluorescent protein variants and quantitative analyses of their self‐assembly process. Sci Rep. 2018;8(1):5344. 29593344 10.1038/s41598-018-23625-7 PMC5871787

[pro70119-bib-0025] Liang M , Li Z , Wang W , Liu J , Liu L , Zhu G , et al. A CRISPR‐Cas12a‐derived biosensing platform for the highly sensitive detection of diverse small molecules. Nat Commun. 2019;10(1):3672. 31413315 10.1038/s41467-019-11648-1 PMC6694116

[pro70119-bib-0026] Majithia R , Patterson J , Bondos SE , Meissner KE . On the design of composite protein–quantum dot biomaterials via self‐assembly. Biomacromolecules. 2011;12(10):3629–3637. 21892824 10.1021/bm200889k

[pro70119-bib-0027] Megley CM , Dickson LA , Maddalo SL , Chandler GJ , Zimmer M . Photophysics and dihedral freedom of the chromophore in yellow, blue, and green fluorescent protein. J Phys Chem B. 2008;113(1):302–308. 10.1021/jp806285s PMC2671006 19067572

[pro70119-bib-0028] Ménard‐Moyon C , Bianco A , Kalantar‐Zadeh K . Two‐dimensional material‐based biosensors for virus detection. ACS Sens. 2020;5(12):3739–3769. 33226779 10.1021/acssensors.0c01961

[pro70119-bib-0029] Mendes GG , Booth RM , Pattison DL , Alvarez AJ , Bondos SE . Generating novel materials using the intrinsically disordered protein Ubx. Methods Enzymol. 2018;611:583–605. 30471701 10.1016/bs.mie.2018.08.007

[pro70119-bib-0043] Mendes GG, Faulk B, Kaparthi B, Irion AR, Fong BL, Bayless K, et al . Genetic functionalization of protein‐based biomaterials via protein fusions. Biomacromolecules. 2024;25(8):4615–5388. 39074364 10.1021/acs.biomac.4c00188 PMC11323028

[pro70119-bib-0030] Nguyen PQ , Silberg JJ . A selection that reports on protein–protein interactions within a thermophilic bacterium. Protein Eng Des Sel. 2010;23(1):529–536. 20418388 10.1093/protein/gzq024 PMC2920301

[pro70119-bib-0031] Patterson JL , Abbey CA , Bayless KJ , Bondos SE . Materials composed of the Drosophila melanogaster protein Ultrabithorax are cytocompatible. J Biomed Mater Res A. 2013;102(1):97–104. 23596050 10.1002/jbm.a.34675

[pro70119-bib-0032] Patterson JL , Arenas‐Gamboa AM , Wang T‐Y , Hsiao H‐C , Howell DW , Pellois JP , et al. Materials composed of the *Drosophila* Hox protein Ultrabithorax are biocompatible and nonimmunogenic. J Biomed Mater Res A. 2014;103(4):1546–1553. 25087647 10.1002/jbm.a.35295

[pro70119-bib-0033] Quijano‐Rubio A , Yeh HW , Park J , Lee H , Langan RA , Boyken SE , et al. De novo design of modular and tunable protein biosensors. Nature. 2021;591(7850):482–487. 33503651 10.1038/s41586-021-03258-z PMC8074680

[pro70119-bib-0034] Romei MG , Boxer SG . Split green fluorescent proteins: scope, limitations, and outlook. Annu Rev Biophys. 2019;48(1):19–44. 30786230 10.1146/annurev-biophys-051013-022846 PMC6537611

[pro70119-bib-0035] Rosenman DJ , Huang Y‐M , Xia K , Fraser K , Jones VE , Lamberson CM , et al. Green‐lighting green fluorescent protein: faster and more efficient folding by eliminating a cis‐trans peptide isomerization event. Protein Sci. 2014;23(4):400–410. 24408076 10.1002/pro.2421 PMC3970891

[pro70119-bib-0036] Sadeghzadeh J , Shahabi P , Farhoudi M , Ebrahimi‐Kalan A , Mobed A , Shahpasand K . Tau protein biosensors in the diagnosis of neurodegenerative diseases. Adv Pharm Bull. 2023;13(3):502–511. 37646056 10.34172/apb.2023.061 PMC10460811

[pro70119-bib-0037] Sandison DR , Webb WW . Background rejection and signal‐to‐noise optimization in confocal and alternative fluorescence microscopes. Appl Optics. 1994;33(4):603–615. 10.1364/AO.33.000603 20862055

[pro70119-bib-0038] Thatikonda N , Nilebäck L , Kempe A , Widhe M , Hedhammar M . Bioactivation of spider silk with basic fibroblast growth factor for in vitro cell culture: a step toward creation of artificial ECM. ACS Biomater Sci Eng. 2018;4(9):3384–3396. 33435072 10.1021/acsbiomaterials.8b00844

[pro70119-bib-0039] Tsai S‐P , Howell DW , Huang Z , Hsiao H‐C , Lu Y , Matthews KS , et al. The effect of protein fusions on the production and mechanical properties of protein‐based materials. Adv Funct Mater. 2015;25(9):1442–1450.

[pro70119-bib-0040] Vivian JT , Callis PR . Mechanisms of tryptophan fluorescence shifts in proteins. Biophys J. 2001;80(5):2093–2109. 11325713 10.1016/S0006-3495(01)76183-8 PMC1301402

[pro70119-bib-0041] Yu TG , Lee J , Yoon J , Choi JM , Kim DG , Heo WD , et al. Engineering of a fluorescent protein for a sensing of an intrinsically disordered protein through transition in the chromophore state. J Am Chem Soc. 2023;3(11):3055–3065. 10.1021/jacsau.3c00445 PMC10685427 38034956

[pro70119-bib-0042] Zhang Q , Chen X , Cui G , Fang W‐H , Thiel W . Concerted asynchronous hula‐twist photoisomerization in the S65T/H148D mutant of green fluorescent protein. Angew Chem Int ed Engl. 2014;53(33):8649–8653. 25044736 10.1002/anie.201405303

