# Supplementary material for: Immobilization and enhancement of a heterodimeric fluorescence biosensor in fibrous protein biomaterials
Source: Protein Sci. 2025 Apr 22;34(5):e70119. doi: 10.1002/pro.70119 (PMC12012991; doi:10.1002/pro.70119)
Supplement: Supplementary file 1 — Data S1. Supporting Information. [file PRO-34-e70119-s001.docx]

**Immobilization and enhancement of a heterodimeric fluorescent biosensor in fibrous protein biomaterials**

Rebecca M. Booth, Amanda Jons, Xue Gong, Shounak Banerjee, Britt Faulk, Hays Rye, Christopher Bystroff, Sarah E. Bondos

**Supplementary Material Tables**

**Supplementary Material Table S1. Protein sequences of constructs as used in experiments.** Amino acid linkers are designated in bold and stop codons are represented by an asterisk. The pCDFDuet plasmid used for expressing both LOO8 and s8 has multiple reading frames by which to produce multiple products at once. Multiple cloning site 1 (MCS1) contains LOO8 or LOO8-Ubx while multiple cloning site 2 (MCS2) contains s8 (underlined) fused C-terminally to the carrier protein Ssp-DnaB mini-intein.

| Ubx1a protein sequence | HHHHHHHHHHSSGHIDDDDKHMGSGSMNSYFEQASGFYGHPHQATGMAMGSGGHHDQTASAAAAAYRGFPLSLGMSPYANHHLQRTTQDSPYDASITAACNKIYGDGAGAYKQDCLNIKADAVNGYKDIWNTGGSNGGGGGGGGGGGGGAGGTGGAGNANGGNAANANGQNNPAGGMPVRPSACTPDSRVGGYLDTSGGSPVSHRGGSAGGNVSVSGGNGNAGGVQSGVGVAGAGTAWNANCTISGAAAQTAAASSLHQASNHTFYPWMAIAGKIRSDLTQYGGISTDMGKRYSESLAGSLLPDWLGTNGLRRRGRQTYTRYQTLELEKEFHTNHYLTRRRRIEMAHALCLTERQIKIWFQNRRMKLKKEIQAIKELNEQEKQAQAQKAAAAAAAAAAVQGGHLDQ* |
| --- | --- |
| LOO8 protein sequence | MCS-1  HHHHHHSSGEDGSVQLADHYQQNTPIGDGPVLLPDNHYLSTQTVLSKDPNEKRDHMVLLEFVTAAGITHGMDELYK**GGTGGS**MASKGEELFTGVVPILVELDGDVNGHKFSVRGEGEGDATIGKLTLKFICTTGKLPVPWPTLVTTLTYGVQCFSRYPDHMKRHDFFKSAMPEGYVQERTISFKDDGKYKTRAVVKFEGDTLVNRIELKGTDFREDGNILGHKLEYNFNSHNVYITADKQKGS*  MCS-2  MKLESAKVSRVFCTGKKLVYILKTRLGRTIKATANHRFLTIDGWKRLDELSLKEHIALPRRLESSSLQLSPEIEKLSQSDIYWDSIVSITETGVEEVFDLTVPGPHNFVANDIIVHNGRSGGTGGNGIKANFTVRHNV* |
| LOO8-Ubx protein sequence | HHHHHHHHHHSSGHEDGSVQLADHYQQNTPIGDGPVLLPDNHYLSTQTVLSKDPNEKRDHMVLLEFVTAAGITHGMDELYK**GGTGGS**MASKGEELFTGVVPILVELDGDVNGHKFSVRGEGEGDATIGKLTLKFICTTGKLPVPWPTLVTTLTYGVQCFSRYPDHMKRHDFFKSAMPEGYVQERTISFKDDGKYKTRAVVKFEGDTLVNRIELKGTDFKEDGNILGHKLEYNFNSHNVYITADKQK**GS**NSYFEQASGFYGHPHQATGMAMGSGGHHDQTASAAAAAYRGFPLSLGMSPYANHHLQRTTQDSPYDASITAACNKIYGDGAGAYKQDCLNIKADAVNGYKDIWNTGGSNGGGGGGGGGGGGGAGGTGGAGNANGGNAANANGQNNPAGGMPVRPSACTPDSRVGGYLDTSGGSPVSHRGGSAGGNVSVSGGNGNAGGVQSGVGVAGAGTAWNANCTISGAAAQTAAASSLHQASNHTFYPWMAIAGKIRSDLTQYGGISTDMGKRYSESLAGSLLPDWLGTNGLRRRGRQTYTRYQTLELEKEFHTNHYLTRRRRIEMAHALCLTERQIKIWFQNRRMKLKKEIQAIKELNEQEKQAQAQKAAAAAAAAAAVQGGHLDQ* |
| S8 peptide | NGIKANFTVRHNV |
| EGFP-Ubx protein sequence | HHHHHHHHHHSSGHIDDDDKHMVSKGEELFTGVVPILVELDGDVNGHKFSVSGEGEGDATYGKLTLKFICTTGKLPVPWPTLVTTLTYGVQCFSRYPDHMKQHDFFKSAMPEGYVQERTIFFKDDGNYKTRAEVKFEGDTLVNRIELKGIDFKEDGNILGHKLEYNYNSHNVYIMADKQKNGIKVNFKIRHNIEDGSVQLADHYQQNTPIGDGPVLLPDNHYLSTQSALSKDPNEKRDHMVLLEFVTAAGITLGMDELYK**GSGSHMGSGS**MNSYFEQASGFYGHPHQATGMAMGSGGHHDQTASAAAAAYRGFPLSLGMSPYANHHLQRTTQDSPYDASITAACNKIYGDGAGAYKQDCLNIKADAVNGYKDIWNTGGSNGGGGGGGGGGGGGAGGTGGAGNANGGNAANANGQNNPAGGMPVRPSACTPDSRVGGYLDTSGGSPVSHRGGSAGGNVSVSGGNGNAGGVQSGVGVAGAGTAWNANCTISGAAAQTAAASSLHQASNHTFYPWMAIAGKIRSDLTQYGGISTDMGKRYSESLAGSLLPDWLGTNGLRRRGRQTYTRYQTLELEKEFHTNHYLTRRRRIEMAHALCLTERQIKIWFQNRRMKLKKEIQAIKELNEQEKQAQAQKAAAAAAAAAAVQGGHLDQ* |

**Supplementary Material Figures**


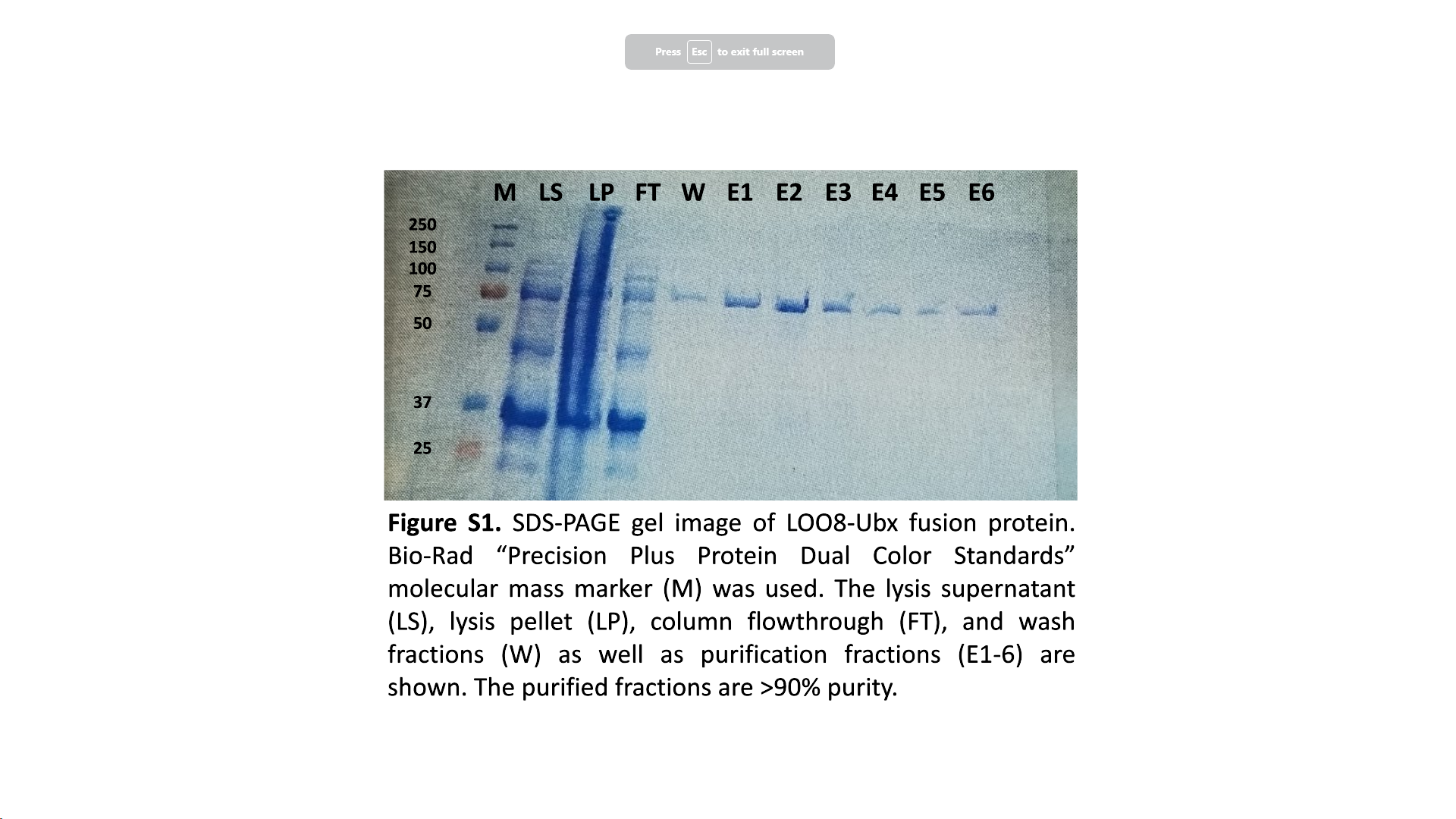


**Supplementary Material Figure S1. SDS-PAGE gel image of LOO8-Ubx fusion protein.** Bio-Rad “Precision Plus Protein Dual Color Stanadards” molecular mass marker (M) was used. The sample lanes were derived from lysis supernatant (LS), lysis pellet (LP), Ni-NTA column flowthrough (FT), and wash fractions (W) as well as purification fractions (E1-6) are shown. The LOO8-Ubx fractions are >90% pure.
